# Comparison of ocular biometric measurements in patients with cataract using three swept-source optical coherence tomography devices

**DOI:** 10.1186/s12886-021-01826-5

**Published:** 2021-01-27

**Authors:** Richul Oh, Joo Youn Oh, Hyuk Jin Choi, Mee Kum Kim, Chang Ho Yoon

**Affiliations:** 1 Department of Ophthalmology, Seoul National University Hospital, 101 Daehak-ro, Jongno-gu, Seoul, 03080 South Korea; 2 Department of Ophthalmology, Seoul National University College of Medicine, Seoul, South Korea; 3 Laboratory of Ocular Regenerative Medicine and Immunology (LORMI), Artificial Eye Center, Seoul National University Hospital Biomedical Research Institute, Seoul, South Korea; 4 Department of Ophthalmology, Seoul National University Hospital Healthcare System Gangnam Center, Seoul, South Korea

**Keywords:** Ocular biometry, Cataract, Total keratometry, Swept-source optical coherence tomography devices, ANTERION, CASIA2, IOLMaster 700

## Abstract

**Background:**

Precise measurement of ocular biometry is critical for determining intraocular lens power. Newly developed swept-source optical coherence tomography (SS-OCT) - based ocular biometric devices, ANTERION and CASIA2 provide ocular biometric measurements as IOLMaster 700. This study aimed to assess agreement between three devices.

**Methods:**

This retrospective comparative study includes patients with cataract who underwent ocular biometric measurements with three devices, ANTERION, CASIA2, and IOLMaster 700, at Seoul National University Hospital, in April 2020. Anterior keratometry, total keratometry, central corneal thickness (CCT), anterior chamber depth (ACD), lens thickness (LT), and axial length (AL) were the main parameters for the comparison. To assess the agreement between the devices, intraclass coefficient (ICC) and Bland-Altman analysis with 95% limits of agreement (LoA) were used.

**Results:**

A total of 47 eyes of 29 patients were measured with three devices. Average anterior keratometry showed excellent agreement (ICC ≥ 0.989), and the mean difference was less than 0.1 D. However, the ICC of the total average keratometry ranged from 0.808 to 0.952, and the difference was more than 0.43 D. The AL measured by ANTERION and IOLMaster 700 showed excellent agreement (ICC = 0.999), and the mean difference was 0.005 mm. The ANTERION and IOLMaster 700 did not obtain AL in six (12.8%) and three (6.4%) cases, respectively (*P* = 0.001 by Fisher’s exact test). The CCT, ACD, and LT also showed excellent agreement (ICC > 0.9).

**Conclusions:**

The new SS-OCT-based devices, ANTERION, and CASIA2 showed a good agreement with IOLMaster 700 in measuring ocular biometry except for the total keratometry. The AL of ANTERION and IOLMaster 700 showed excellent agreement.

## Background

In modern cataract surgery, it is important not only to remove the cataract but also to achieve accurate postoperative refractive error. The higher the generation of the intraocular lens (IOL) calculation formula, the higher the accuracy, and more ocular biometric parameters are required [1]. In this regard, precise measurement of ocular biometry is critical for determining the power of IOLs [2]. Recently, ocular biometry measurement devices with principles of swept-source optical coherence tomography (SS-OCT) were developed. SS-OCT devices use 1000–1350 nm of wavelength. They can provide a whole image from the cornea to the posterior lens. They are known to have a superior ability to successfully measure the axial length (AL) compared with a partial coherence interferometry device [3, 4]. IOLMaster 700 (Carl Zeiss Meditec), the first SS-OCT-based biometric device, is one of the most widely used devices for cataract surgery [3]. Many studies have shown that IOLMaster 700 had good agreement with other devices, including IOLMaster 500 [5–7].

ANTERION (Heidelberg Engineering) and CASIA2 (Tomey) are newly developed SS-OCT devices. ANTERION uses a 1300 nm central wavelength of light. It provides a scan depth range of 32 mm for the axial length and an in-tissue axial resolution of < 10 μm. It uses OCT-based structural images to generate ocular biometric measurements [8]. CASIA2, an advanced version of CASIA SS-1000, uses a 1310 nm wavelength light with a scan speed of 50,000 A scans per second. CASIA2 has a scan range of 13 mm depth and 16 mm width [9]. They both provide ocular biometric measurements, including the anterior chamber depth (ACD), lens thickness (LT), and corneal keratometry. In addition, ANTERION with its deeper scan range, provides measurement of AL. They generally showed good repeatability and agreement with the IOLMaster 700 device [8–11]. However, so far, no previous studies have compared ANTERION and CASIA2 or all three devices in patients with cataract. This study aimed to assess the agreement between three devices, ANTERION, CASIA2, and IOLMaster 700, in terms of ocular biometry.

## Methods

This retrospective study comprised patients with cataracts from Seoul National University Hospital (SNUH), Seoul, Republic of Korea, in April 2020. All procedures were conducted following the tenets of the Declaration of Helsinki, and the study design was approved by the Institutional Review Board of SNUH (IRB No. 2006-026-1130). Owing to the retrospective design of the study and the use of deidentified patient information, the review board waived the need for written informed consent.

All patients underwent a standard test for cataract surgery according to the SNUH preoperative cataract examination protocol. The standard test for cataract surgery includes measurements using the three SS-OCT devices, specular microscopy, macular OCT, corneal topography, ultrasound A-scan, and automated keratometry. Among them, optical biometric measurements using the three SS-OCT devices were conducted before the other examinations. Patients who were diagnosed with retinal diseases, such as epiretinal membrane, age-related macular degeneration, or corneal diseases such as corneal opacity, keratoconus, and pterygium were excluded from this study. Patients who had already undergone refractive surgery were also excluded. Ocular biometry measurements, including both anterior and total keratometry, central corneal thickness (CCT), ACD, and LT, were obtained by ANTERION, CASIA2, and IOLMaster 700. AL was measured only by ANTERION and IOLMaster 700 because CASIA2 does not provide AL measurement. ACD was defined as the axial distance from the corneal endothelium to the lens. Because IOLMaster 700 measures the ACD from the corneal epithelium to the anterior lens surface, we subtracted the CCT value from the ACD measured by the IOLMaster 700.

Ocular biometric evaluation proceeded in the order of ANTERION, CASIA2, and IOLMaster 700. Measurements were performed by one examination specialist. The three devices were lined up in the same examination room. We confirmed that every time we examined the patient, the room was constantly illuminated under 10 lx, as measured with a light meter (LX-1102, Lutron, Taiwan).

All statistical analyses were performed using R software (R version 4.0.2. Available at http://www.r-project.org; accessed June 2020). Continuous variables are presented as mean ± standard deviation. Acquisition rates of AL measurements were analyzed by Fisher’s exact test. To assess the agreement between the measurements of the devices, intraclass correlation coefficients (ICC, two-way random, single measure) were calculated [12]. ICC was regarded as follows: < 0.75, poor to moderate reliability; 0.75–0.90, good reliability; and > 0.90, excellent reliability [11]. Bland-Altman analysis with 95% limits of agreement (LoA) was also used for all pairs. The level of statistical significance was set at *P* < .05. Due to the retrospective design of this study, the sample size was not calculated.

## Results

**Fig. 1:**
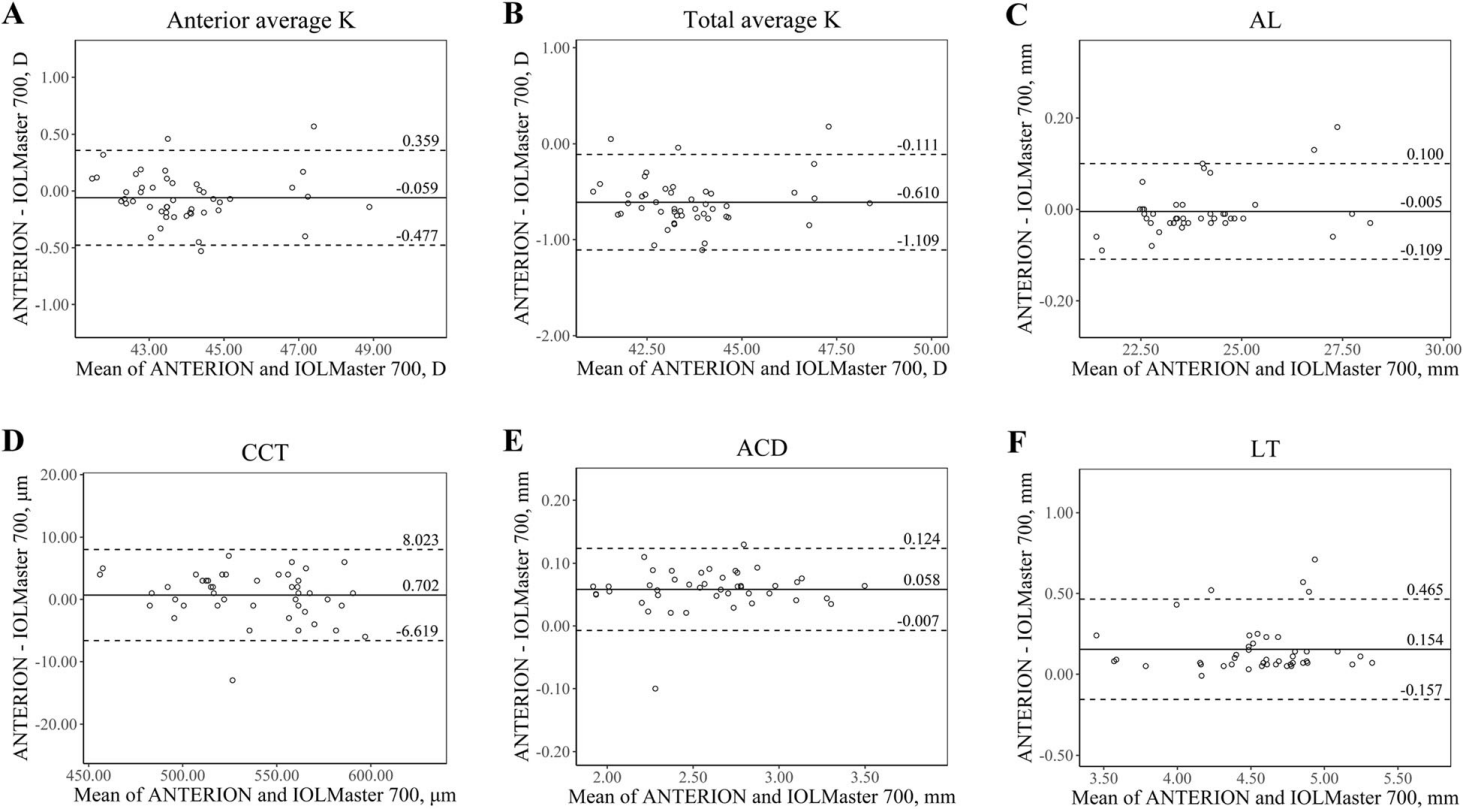
The Bland-Altman plots show agreement between parameters measured by ANTERION and IOLMaster 700. The solid lines show the mean differences, and the dotted lines show the lower and upper 95% LoA. **a** Anterior average K, **b** Total average K, **c** AL, **d** CCT, **e** ACD, **f** LT. (ACD = anterior chamber depth; AL = axial length; CCT = central corneal thickness; D = diopter; K = keratometry; LT = lens thickness)

**Fig. 2:**
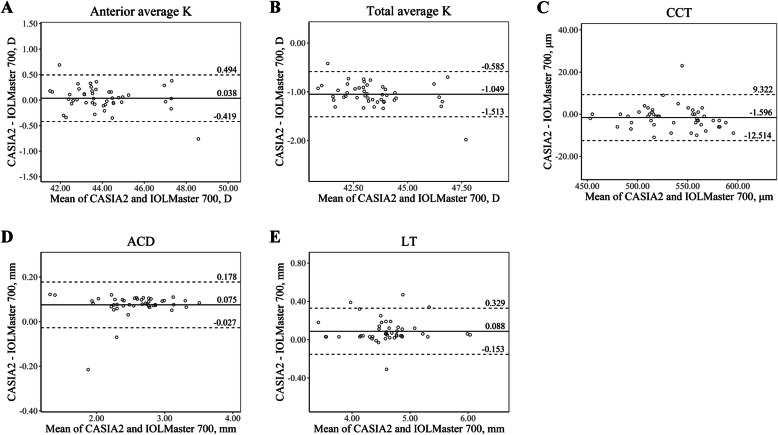
The Bland-Altman plots show agreement between parameters measured by CASIA2 and IOLMaster 700. The solid lines show the mean differences, and the dotted lines show the lower and upper 95% LoA. **a** Anterior average K, **b** Total average K, **c** CCT, **d** ACD, **e** LT. (ACD = anterior chamber depth; CCT = central corneal thickness; D = diopter; K = keratometry; LT = lens thickness)

**Fig. 3:**
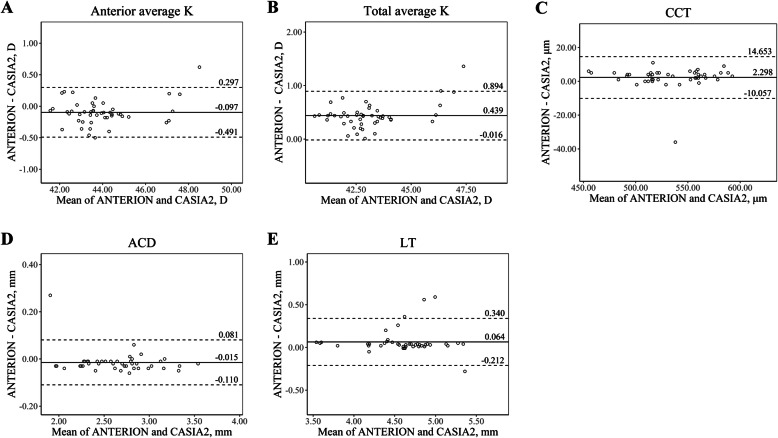
The Bland-Altman plots show agreement between parameters measured by ANTERION and CASIA2. The solid lines show the mean differences, and the dotted lines show the lower and upper 95% LoA. **a** Anterior average K, **b** Total average K, **c** CCT, **d** ACD, **e** LT. (ACD = anterior chamber depth; CCT = central corneal thickness; D = diopter; K = keratometry; LT = lens thickness)

A total of 47 eyes of 29 patients with cataract (14 males, 15 females) were included in this study, and their mean age was 64.41 ± 10.68 (range, 31–83 years). Table 1 shows the descriptive summary of the ocular biometric measurements taken by the three devices and their ICC values. For most of the parameters, ICC values between the devices were greater than 0.90. Bland-Altman plots for agreement analysis between the devices are presented in Figs. 1, 2 and 3 (Fig. 1, ANTERION vs. Master 700; Fig. 2, CASIA2 vs. Master 700; Fig. 3, ANTERION vs. CASIA2).

**Table 1:** Summary of the ocular biometric measurements measured by ANTERION, CASIA2, and IOLMaster 700

	ANTERION	CASIA2	IOLMaster 700	ANTERION vs IOLMaster 700		CASIA2 vs IOLMaster 700		ANTERION vs CASIA2	
				ICC	Difference	ICC	Difference	ICC	Difference
Anterior average K, D	43.92 ± 1.62	44.02 ± 1.57	43.98 ± 1.63	0.991	− 0.059 ± 0.213	0.989	0.038 ± 0.233	0.990	− 0.097 ± 0.201
Anterior steep K, D	44.42 ± 1.62	44.51 ± 1.55	44.38 ± 1.60	0.974	0.034 ± 0.367	0.959	0.13 ± 0.439	0.962	−0.096 ± 0.433
Anterior flat K, D	43.42 ± 1.70	43.53 ± 1.66	43.58 ± 1.68	0.976	−0.166 ± 0.334	0.978	−0.059 ± 0.348	0.970	−0.107 ± 0.403
Anterior cylinder K, D	1.01 ± 0.71	0.99 ± 0.76	0.80 ± 0.46	0.598	0.21 ± 0.516	0.443	0.189 ± 0.653	0.526	0.021 ± 0.719
Total average K, D	43.33 ± 1.63	42.89 ± 1.52	43.94 ± 1.61	0.922	−0.61 ± 0.255	0.808	− 1.049 ± 0.237	0.952	0.439 ± 0.232
Total steep K, D	43.85 ± 1.64	43.36 ± 1.57	44.37 ± 1.64	0.920	−0.525 ± 0.425	0.801	− 1.016 ± 0.443	0.918	0.491 ± 0.453
Total flat K, D	42.81 ± 1.69	42.42 ± 1.56	43.51 ± 1.62	0.895	−0.708 ± 0.359	0.790	− 1.094 ± 0.353	0.938	0.386 ± 0.438
Total cylinder K, D	1.04 ± 0.74	0.94 ± 0.73	0.86 ± 0.47	0.505	0.183 ± 0.602	0.441	0.078 ± 0.649	0.461	0.105 ± 0.761
AL^a^, mm	24.00 ± 1.60	NA	23.99 ± 1.54	0.999	−0.005 ± 0.053	NA	NA	NA	NA
CCT, μm	535.89 ± 34.29	533.60 ± 34.26	535.19 ± 35.04	0.994	0.702 ± 3.735	0.986	−1.596 ± 5.57	0.981	2.298 ± 6.304
ACD, mm	2.62 ± 0.39	2.60 ± 0.48	2.53 ± 0.47	0.990	0.058 ± 0.033	0.982	0.075 ± 0.052	0.994	− 0.015 ± 0.049
LT^b^, mm	4.63 ± 0.43	4.60 ± 0.55	4.54 ± 0.52	0.915	0.154 ± 0.159	0.963	0.088 ± 0.123	0.961	0.064 ± 0.141

Continuous data are presented as mean ± standard deviation

Differences between two devices were defined by subtracting the latter value from the former value

*ACD* anterior chamber depth, *AL* axial length, *CCT* central corneal thickness, *D* Diopter, *ICC* intraclass correlation coefficient, *K* keratometry, *LT* lens thickness, *NA* not available, *vs* versus

^a^AL was analyzed with 41 eyes that could be measured by both ANTERION and IOLMaster 700

^b^LT was analyzed with 44 eyes that could be measured by all three devices

In the measurement of anterior keratometry, ANTERION and CASIA2 showed excellent agreement with IOLMaster 700 (ICC value of 0.991, and 0.989, respectively). Anterior keratometry measured by ANTERION and CASIA2 had an ICC value of 0.990. The mean differences were less than 0.060 D.

In the measurement of total keratometry, ANTERION and CASIA2 had the greatest ICC values (0.952). While ANTERION and IOLMaster 700 had an ICC value of 0.922, CASIA2 and IOLMaster 700 had an ICC value of 0.808. The mean difference in total keratometry between CASIA2 and IOLMaster 700, ANTERION and IOLMaster 700, and ANTERION and CASIA2 was 1.049 D, 0.61 D, and 0.439 D, respectively.

AL measurements were not obtained in three eyes with the IOLMaster 700 and six eyes with the ANTERION (*P* = 0.001 by Fisher’s exact test). Both IOLMaster 700 and ANTERION failed to measure AL in three eyes. Lens grading of these three eyes were intumescent, NO6, and P5 according to the LOCS III classification [13]. In addition, ANTERION also failed to measure AL in other three eyes, of which ALs were successfully obtained by IOLMaster 700. The lens grade of these eyes was C1NO1, P4, and anterior subcapsular opacity (ASC).

In the measurement of ACD, new SS-OCT devices, ANTERION, and CASIA2 had the greatest ICC value (0.994). Each of them had a comparably great agreement with the IOLMaster 700 (ICC value of 0.990, 0.982, relatively).

CCT and ACD also showed excellent agreement among the three devices. The mean differences of CCT and ACD between the devices were less than 3 μm and 0.08 mm, respectively. The Bland-Altman plot of CCT and ACD showed that most differences were within 10 μm and 0.15 mm, respectively.

In the measurement of LT, CASIA2 measured LT in all eyes. However, ANTERION and IOLMaster 700 failed to measure LT in four and two eyes, respectively. For one eye with ASC, both ANTERION and IOLMaster 700 failed to measure LT. With the eyes with measurable LT in all devices, they also showed excellent agreement with each other, as shown in Table 1.

## Discussion

In this study, we evaluated the agreement of ocular biometric measurements in patients with cataracts among the three SS-OCT devices. To the best of our knowledge, this is the first study to compare the three devices simultaneously. In addition, for the first time, we compared the total corneal power values that account for posterior corneal curvature.

Generally, anterior keratometry measurements showed excellent agreement. We found the difference of anterior average K of each device was less than 0.1 D (ANTERION vs. IOLMaster 700, − 0.059 D; CASIA2 vs. IOLMaster 700, 0.038 D; and ANTERION vs. CASIA2, − 0.097 D), which seems to be clinically insignificant. This is in line with the study by Fisus et al. [8] that the mean absolute difference was 0.04 D when comparing ANTERION and IOLMaster 700.

Regarding total keratometry, a better refractive outcome was achieved using total keratometry compared to conventional anterior keratometry in the calculation of IOLs for cataract surgery by IOLMaster 700 [14–16]. In addition, it is expected that more patients will undergo cataract surgery who have previously undergone refractive surgery; therefore, it will become more important to accurately measure total corneal power [15, 17]. However, the total average keratometry measured by the three devices showed a difference of 0.439 D or more. ICC values for total average keratometry were lower than those of anterior average keratometry, and the Bland-Altman plot of total average keratometry showed a wider 95% LoA than that of average anterior keratometry. The corneal power difference of 0.439 D is about 0.64 D in the IOL plane, assuming the IOL plane to the corneal plane equivalent to the power conversion factor is about 0.69 [18]. Considering that the 0.5 D is the currently used IOL power step, the total keratometry values derived from each device should not be interchanged when calculating IOLs. The discrepancy of the three devices in total keratometry could be caused by the different algorism for calculating total keratometry in each device [5, 8, 19]. IOLMaster 700 obtains anterior keratometry with 18 reflected spots in hexagonal patterns at three zones (1.5 mm, 2.5 mm, and 3.5 mm) and posterior keratometry with SS-OCT tomography [16, 20]. Meanwhile, ANTERION and CASIA2 calculate anterior and total keratometry only with SS-OCT images (ANTERION: 65 B-scans with 256 A-scans each; CASIA2: 16 B-scans with 800 A-scans each) [8, 21].

We compared AL measured by only ANTERION with IOLMaster 700, because CASIA2 does not provide AL. It is known that the interdevice agreement of AL measured by optical method is excellent [22]. We also found ANTERION with IOLMaster 700 had an excellent agreement in measuring AL, with an ICC value of 0.999, and the average difference was 0.005 mm. Accounting that a measurement error of 1 mm of AL induces 2.5 D deviation in IOL calculation in the eye with an average AL (23.5 mm), those 0.005 mm of AL difference result in 0.0125 D of IOL power difference, and it seems that the AL by ANTERION and IOL Master 700 could be interchangeable [23]. AL measurement by optical biometry has been shown to fail in eyes with dense or posterior capsular cataract [5]. In this study, AL of six and three eyes were not obtained by ANTERION and IOLMaster 700, respectively, and most of their cataract status was dense nucleus cataract or posterior or anterior subcapsular cataract. ANTERION showed significantly higher AL measurement failure than IOLMaster 700. This result was unexpected because longer wavelengths improve penetration, and ANTERION (1300 nm) uses a longer wavelength than IOLMaster 700 (1050 nm) for measuring AL [24]. The different acquisition methods could be the cause of this discrepancy. IOLMaster 700 measures AL by the average values of three scans in each of the six meridians [5]. ANTERION obtains AL by averaging three consecutive three subsets of data.

In the measurement of CCT, ACD, and LT, the three devices showed excellent agreement. The 95% LoAs were narrow and clinically insignificant. Regarding CCT, it is also important for screening before refractive surgery. In our study, which included only normal corneas, CCT seems to be interchangeable; however, further study is needed for pathologic conditions such as keratoconus or post-refractive surgery patients.

This study has several limitations. First, the sample size was relatively small. Second, all included patients who had cataracts. Since cataracts can affect optical physics during measurements, there might be some biases. Further studies with normal eyes will provide more information about the agreements between these devices. Third, all patients were Asian. Therefore, our data may not be generalizable to other ethnicities. Finally, we did not use a randomization sequence when measuring the three machines. However, they all are non-contact devices, and it can be assumed that the order of measurements would not affect ocular biometric values.

## Conclusions

SS-OCT devices, ANTERION, CASIA2, and IOL Master 700 showed good agreement in parameters of anterior corneal curvature, CCT, ACD, and LT. AL of ANTERION and IOLMaster 700 showed excellent agreement, and it seems to be interchangeable. However, the total keratometry value of each device was different and should not be used interchangeably.

## Data Availability

The datasets used and analyzed during the current study are available from the corresponding author on reasonable request.

## Abbreviations

ACD: Anterior chamber depth
ASC: Anterior subcapsular opacity
AL: Axial length
CCT: Central corneal thickness
ICC: Intraclass correlation coefficients
IOL: Intraocular lens
LT: Lens thickness
LoA: Limits of agreement
SS-OCT: Swept-source optical coherence tomography
